# Trehalose Treatment in Zebrafish Model of Lafora Disease

**DOI:** 10.3390/ijms23126874

**Published:** 2022-06-20

**Authors:** Stefania Della Vecchia, Asahi Ogi, Rosario Licitra, Francesca Abramo, Gabriele Nardi, Serena Mero, Silvia Landi, Roberta Battini, Federico Sicca, Gian Michele Ratto, Filippo Maria Santorelli, Maria Marchese

**Affiliations:** 1 Department of Developmental Neuroscience, IRCCS Fondazione Stella Maris, 56128 Pisa, Italy; stefania.dellavecchia@fsm.unipi.it (S.D.V.); roberta.battini@fsm.unipi.it (R.B.); 2 Department of Clinical and Experimental Medicine, University of Pisa, 56126 Pisa, Italy; 3 Department Neurobiology and Molecular Medicine, IRCCS Fondazione Stella Maris, 56128 Pisa, Italy; a.ogi@hotmail.com (A.O.); rosario.licitra@fsm.unipi.it (R.L.); serena.mero@fsm.unipi.it (S.M.); 4 Department of Veterinary Science, University of Pisa, 56124 Pisa, Italy; francesca.abramo@unipi.it; 5 National Enterprise for Nanoscience and Nanotechnology (NEST), Consiglio Nazionale delle Ricerche (CNR)-Istituto Nanoscienze and Scuola Normale Superiore Pisa, 56127 Pisa, Italy; gabriele.nardi@sns.it (G.N.); silvia.landi@in.cnr.it (S.L.); gianmichele.ratto@sns.it (G.M.R.); 6 Consiglio Nazionale delle Ricerche (CNR)-Istituto di Neuroscienze, 56124 Pisa, Italy; 7 Child Neuropsychiatric Unit, USL Centro Toscana, 59100 Prato, Italy; federico.sicca@gmail.com

**Keywords:** Lafora disease, progressive myoclonic epilepsies, neuroinflammation, autophagy, trehalose

## Abstract

Mutations in the *EPM2A* gene encoding laforin cause Lafora disease (LD), a progressive myoclonic epilepsy characterized by drug-resistant seizures and progressive neurological impairment. To date, rodents are the only available models for studying LD; however, their use for drug screening is limited by regulatory restrictions and high breeding costs. To investigate the role of laforin loss of function in early neurodevelopment, and to screen for possible new compounds for treating the disorder, we developed a zebrafish model of LD. Our results showed the *epm2a^−/−^* zebrafish to be a faithful model of LD, exhibiting the main disease features, namely motor impairment and neuronal hyperexcitability with spontaneous seizures. The model also showed increased inflammatory response and apoptotic death, as well as an altered autophagy pathway that occurs early in development and likely contributes to the disease progression. Early administration of trehalose was found to be effective for rescuing motor impairment and neuronal hyperexcitability associated with seizures. Our study adds a new tool for investigating LD and might help to identify new treatment opportunities.

## 1. Introduction

Lafora disease (LD, OMIM #254780) is a rare autosomal recessive neurodegenerative disorder with a worldwide prevalence of almost four cases per million [1]. LD is associated with mutations in three genes: *EPM2A* [2], encoding laforin, *EPM2B* [3], encoding malin, and *PRMD8* [4], which codes for a protein responsible for the translocation of laforin and malin to the nucleus. However, diagnostic confirmation shows mutations in the *EPM2A* or *EPM2B* gene in more than 95% of diagnosed patients [5,6].

LD manifests between eight and 19 years of age as a form of progressive myoclonic epilepsy (PME) [1] characterized by multiple types of seizures, including tonic-clonic seizures, drop attacks, and action-sensitive and stimulus-sensitive myoclonus [1,7]. Progressive worsening of the seizures typically leads to severe, drug-resistant epilepsy, with possible episodes of status epilepticus [1,5]. In LD patients, EEG recordings typically show slowing of the background activity, diffuse or predominantly posterior paroxysmal abnormalities, and photosensitivity [1,7,8]. Neurological deterioration leads to progressive neuropsychiatric and motor impairments that include behavioral disturbances, cognitive decline up to dementia, cerebellar ataxia, and spasticity [1,9,10]. Death usually occurs at around 10 years from the onset of the disease, usually due to status epilepticus or complications related to degeneration of the nervous system [11].

LD is characterized by the accumulation, over time, of LBs in the cytoplasm of some cells, including neurons [12]. LBs are made of polyglucosan (a hyperphosphorylated, insoluble form of glycogen with abnormally long and poorly branched chains) that precipitates, aggregates, and accumulates [13,14,15]. In addition to the carbohydrate component, LBs contain a small amount of protein, consisting of laforin, autophagy proteins, chaperones, glycogen metabolism enzymes, and others [16,17,18]. Due to the presence of LBs, LD has always been considered a disease of impaired glycogen metabolism. However, many findings indicate a more complex condition, in which the dysfunction of glycogen metabolism is flanked by alterations of different cellular metabolic pathways [19,20], whose causal relationships remain to be defined. In the laforin-deficient mouse model, LBs are accompanied by alterations in the autophagy process [21], increased oxidative stress [22], and neuroinflammation [23,24].

Laforin orthologs are found in all vertebrates and several unicellular eukaryotes, reflecting the fundamental biological role of this protein [25,26]. Laforin together with malin has been implicated in numerous processes: regulation of glycogen synthesis [15], protein quality control by the ubiquitin-proteasome [27,28,29] and autophagy-lysosome systems [21,30,31], oxidative stress [22,32,33], and others [34].

Despite numerous advances, LD remains to date an incurable condition. Targeted therapies for the disease are under investigation, with the aim of preventing the formation of LBs or eliminating their accumulation [19]. As far as new direct therapies aimed at gene replacement therapy are available for humans, the most promising and less invasive approaches are based on small molecules and EMA-FDA-approved repurposed drugs for other clinical conditions [19]. Although there exists a rare canine form of LD [28,29], rodents are usually used for studying the disease pathomechanisms and for drug screening purposes. To date, several drugs have been repurposed for LD and tested in mouse models, such as metformin and 4-phenylbutyric acid [35], sodium selenate [36], dexamethasone [37] and trehalose [38]. Given the growing possibilities of drug repurposing in storage disorders such as LD and the necessity to screen new small molecules, there is a need for feasible and more suitable disease models that allow faster results and reducing the costs for pharmacological testing. In this scenario, the zebrafish (*Danio rerio*) represents an excellent tool to be used alongside the mouse model for high-throughput drug screening. Zebrafish has been used successfully to study neuro-developmental and neurodegenerative disorders, and shows numerous advantages over its murine counterpart, among which certainly worth mentioning are its simple breeding and maintenance requirements, high fertility and rapid external development which allow for the reduction in drug screening time [39,40,41,42]. Therefore, the generation of a zebrafish model can help to define therapeutical compounds that can be transferred to rodents and then potentially tested in patients.

Using CRISPR/Cas9 technology [43], we generated an *epm2a*^−/−^ knock-out zebrafish strain to further investigate the consequences of laforin loss of function on cell metabolism and early neurodevelopment, and to identify a reliable and versatile model for carrying out new drug screenings in this severe metabolic disorder. Our LD zebrafish model recapitulates the human phenotype by showing motor impairment and spontaneous seizures. It also recapitulates [1] several pathological features of LD already demonstrated in mouse models of the disease, such as glycogen accumulation, increased apoptotic cell death, neuroinflammation and autophagy abnormalities. Using trehalose, an autophagy stimulator as an example of one of the repurposed drugs tested in LD [38], we also observed in larvae the rescue of the motor and epileptic impairment and demonstrated that our model represents a valid tool for LD pharmacological screening.

## 2. Results

### 2.1. Regional and Developmental Expression of the epm2a Gene

**Figure 1:**
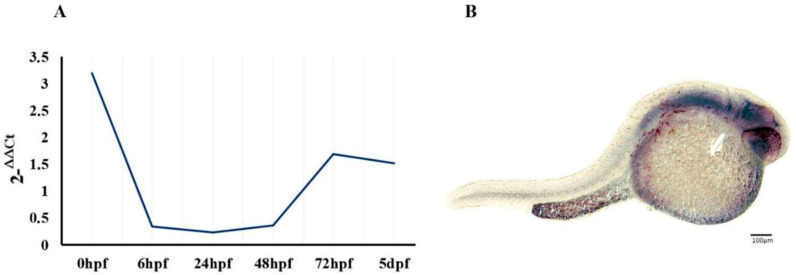
Regional and developmental expression of the *epm2a* gene. (**A**) qRT−PCR analysis showing epm2a developmental relative expression from 0 hpf to 5 dpf in WT zebrafish. Three independent RNA samples (each obtained from about 30–40 larvae) for each stage. (**B**) Wholemount in situ hybridization showing epm2a regional expression in 24 hpf WT embryos.

We investigated the regional and developmental expression of *epm2a* in zebrafish. Developmental expression of the *epm2a* transcript was assessed by qRT-PCR analysis at a series of developmental stages—0 h post-fertilization (hpf), six hpf, 24 hpf, 48 hpf, 72 hpf and five days post-fertilization (dpf)—in WT zebrafish larvae. We observed high levels of *epm2a* expression at 0 hpf that decreased over the next few hours before increasing gradually from 48 hpf (Figure 1A). A similar trend was observed in mouse brains, where levels of Epm2a transcript increased gradually after birth, reaching the highest level in adults [44]. Regional expression of *epm2a* tested in WT zebrafish embryos at 24 hpf showed laforin to be distributed almost ubiquitously within the zebrafish central nervous system (Figure 1B).

### 2.2. Generation of epm2a^−/−^ Mutant Zebrafish

**Figure 2:**
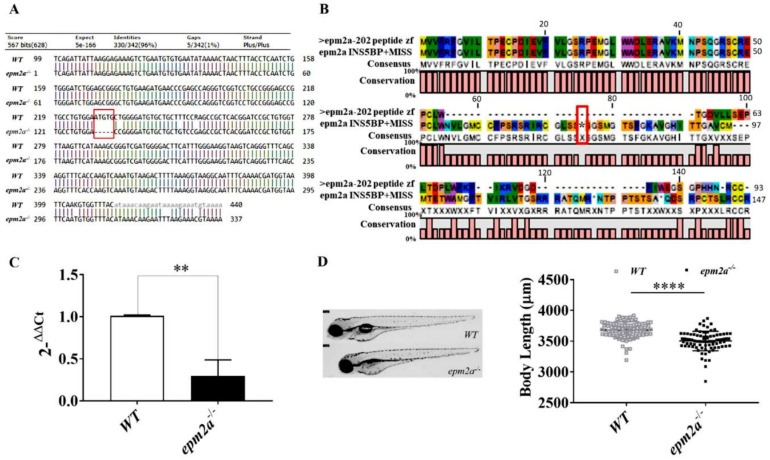
Generation and morphology of *epm2a^−/−^* zebrafish mutants. (**A**) Coding sequence alignment of wt and mutant *epm2a* shows the generation of *epm2a*-null mutant zebrafish by insertion of 5 bp into exon 2 of the *epm2a* gene. (**B**) Protein alignment of wt and mutant *epm2a*. The red rectangle shows the premature stop codon at residue 74 (p.Thr54Asnfs*74) caused by the 5-bp insertion mutation in exon 2. (**C**) qRT–PCR analysis revealed a decrease in the level of *epm2a* mRNA expression, normalized to the expression of *β-actin* mRNA. Three independent RNA samples (each obtained from about 30–40 larvae) from *epm2a^−/−^* larvae at 120 hpf and from controls were analyzed. The values are expressed as mean ± standard deviation (SD). **, *p* ≤ 0.01, calculated by Student’s *t*-test. (**D**) Lateral view photographs of representative control and *epm2a^−/−^* specimens. At 4 dpf we observed a significant reduction in body length in *epm2a^−/−^* larvae (*n* = 83) compared to WT controls (*n* = 83), **** *p* ≤ 0.0001, calculated by Mann-Whitney test.

The zebrafish *epm2a* gene (ENSDARG00000059044), which maps to chromosome 23, consists of five coding exons, and encodes laforin, a 310-amino acid protein. Performing a bioinformatics analysis of the amino acid sequence of Danio rerio and of Homo sapiens, we observed high conservation of the zebrafish protein compared with its human counterpart (75% homology and 62% identity) (Supplementary Figure S1). By means of CRISPR/Cas9 editing, we engineered zebrafish *epm2a*-null mutants in which a 5-bp insertion mutation in exon 2 of the *epm2a* gene (Figure 2A) led to a frameshift mutation and premature stop codon at residue 74 (p.Thr54Asnfs*74) (Figure 2B). The homozygous *epm2a^−/−^* F2 zebrafish line was bred to adulthood, and its progeny were used to study the phenotype of the mutant strain, and to determine whether it replicates the characteristics of LD. Given the lack of a reliable antibody against zebrafish laforin, it was not possible to test protein abundance in the mutant zebrafish strain by western blotting. However, qRT-PCR analysis showed a significant decrease in the amount of *epm2a* transcript in mutants compared with WT siblings at 5 dpf (Figure 2C). Homozygous *epm2a^−/−^* larvae showed grossly normal morphology (Figure 2D) but were significantly different in length from their control siblings (*n* = 83) at 4 dpf. 

### 2.3. Glycogen Accumulation and Lafora Body Formation

**Figure 3:**
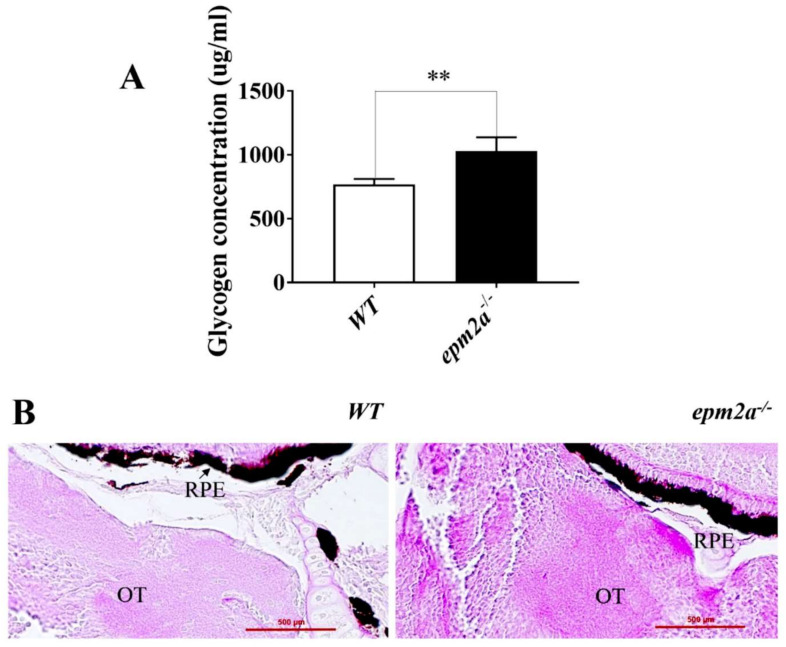
Accumulation of glycogen in *epm2a^−/−^* larvae at 5 dpf. (**A**) Glycogen concentration, expressed in ug/mL, measured in *epm2a^−/−^* larvae (*n* = 30) and WT controls (*n* = 30) at 5 dpf. The values are expressed as mean ± standard deviation (SD). **, *p* ≤ 0.01, calculated by Student’s *t*-test. (**B**) Histological examination with periodic acid-Schiff staining of the brains of *epm2a^−/−^* and WT larvae at five dpf (magnification 40×). Abbreviations: OT, optic tectum; RPE, retinal pigment epithelium.

The hallmark of LD is the accumulation of glycogen and subsequent formation of LBs in several tissues, including the nervous tissue. To assess whether these features are already present in the early stages of the disease, we first tested glycogen accumulation in whole zebrafish larvae at 5 dpf, observing significantly increased glycogen concentration (measured as ug/mL) in *epm2a^−/−^* mutants (*n* = 30) compared with WT larvae (*n* = 30) (*p* < 0.01) (Figure 3A). Then, by means of PAS staining of *epm2a^−/−^* mutants and WT controls, we looked for LBs in zebrafish larvae at 5 dpf. Histological examination with PAS staining did not detect LBs in the brains of the *epm2a*^−/−^ mutants; however, a slight increase in positive PAS staining was observed in mutants compared with WT larvae at 5 dpf (Figure 3B).

### 2.4. Mutant Zebrafish Displays Neuronal Hyperexcitability and Spontaneous Seizures

**Figure 4:**
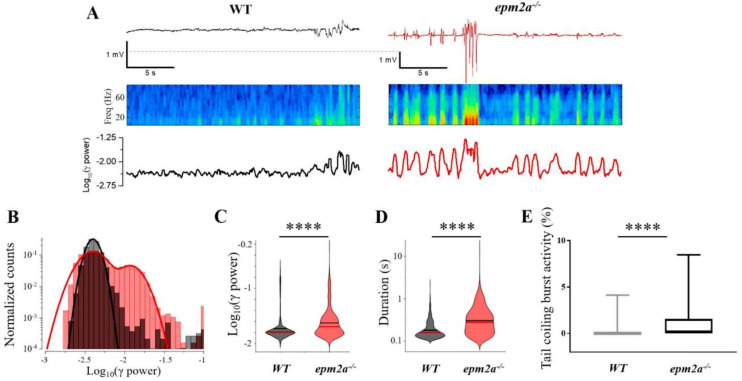
LFP and burst activity recordings in *epm2a^−/−^* zebrafish mutants. (**A**) (Top) Snapshot of 25 s–long extract of LFP signal recorded from 120 hpf WT (left) and *epm2a^−/−^* (right) zebrafish showing differences in burst activity. The relative spectrogram (Middle) and power in the 30–95 Hz band computed on a rolling window of 250 ms in steps of 50 ms (Bottom) are also shown. (**B**) Distribution of the log10 of the power in the 30–95 Hz band from the complete recordings (15 min) of the larvae depicted in panels A and B (WT in black and *epm2a^−/−^* in red). (**C**) and (**D**) Violin plots of the power and duration of the events detected from all the WT (*n* = 11) and *epm2a^−/−^* (*n* = 11) fish. Red and black lines indicate medians and means, respectively. (**E**) Coiling frequency recorded for 60 s at 30 hpf is increased in *epm2a^−/−^* embryos (*n* = 157) with respect to WT controls (*n* = 174). ****: *p* ≤ 0.0001 calculated by Mann-Whitney test.

Forebrain local field potential (LFP) recordings at five dpf showed a higher frequency of high-power electrophysiological events in *epm2a^−/−^* larvae compared with WT siblings. In particular, the duration and power (i.e., RMS power of the signal measured in the 30–95 Hz band, see Section 4) of high-power electrophysiological events were statistically significantly increased in *epm2a^−/−^* larvae compared to WT siblings, likely accounting for their spontaneous seizures (Figure 4A). On further analysis, the high-power events observed in the *epm2a^−/−^* recordings were found to differ in morphology compared with the activity recorded in controls (Figure 4B). Both experimental groups exhibited frequent events characterized by low duration and low power, likely associated with normal brain activity. However, only the mutants showed the seizure-like events associated with multiple high-power, long-duration (>1 s) discharges (Figure 4C,D), as well as smaller events, sometimes associated with interictal-like discharges. The presence of these spontaneous high-power events is an important pathological sign, given that the larvae were recorded in normal conditions and were not challenged with any pro-epileptic stimuli. These events were never observed in the control group. Furthermore, these data were supported by behavioral analysis at 30 hpf, which showed an increase in spontaneous burst activity in knockout embryos compared with WT controls (*p* < 0.0001) (Figure 4E).

### 2.5. Motor Impairment in epm2a^−/−^ Mutant Zebrafish

**Figure 5:**
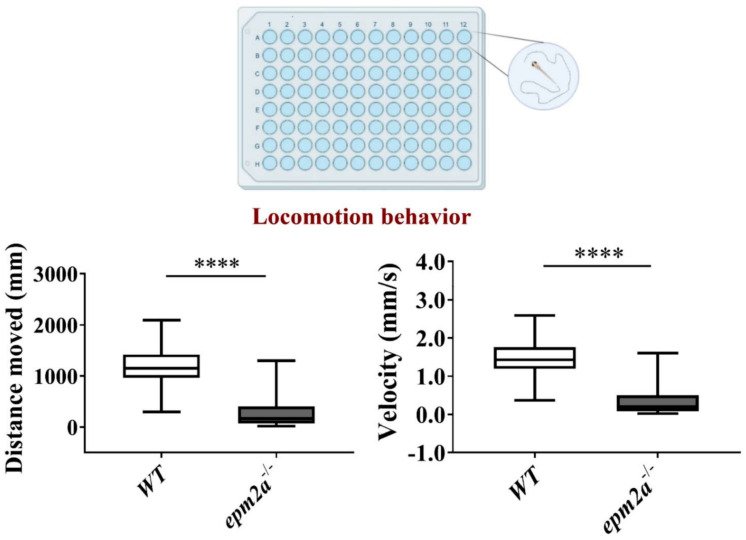
Locomotor activity in *epm2a^−/−^* zebrafish larvae. Automated analysis of spontaneous motor activity recording for 30 min revealed reductions in distance traveled and velocity in *epm2a^−/−^* larvae compared with control siblings at 120 hpf (*epm2a^−/−^ n* = 190; controls *n* = 152, in 3 independent experiments). Statistical analysis (**** *p* ≤ 0.0001) was performed using the Mann-Whitney test.

Whilst their increased burst activity at 30 hpf likely indicates a higher frequency of “seizures”, *epm2a^−/−^* larvae, compared with WT controls, showed significantly reduced motor activity (slowing and reduced distance traveled) at five dpf (*p* < 0.0001) (Figure 5). To rule out morphological alteration of spinal motor neurons as the cause of these events, we used a motor axon marker, synaptotagmin-2, to label and study the motor axons; however, no alterations in arborization or length were seen in *epm2a^−/−^* larvae compared with WT controls (Supplementary Figure S2).

### 2.6. Neuroinflammation, Apoptotic Death, and Mitochondrial Dysfunction in epm2a^−/−^ Zebrafish Larvae

**Figure 6:**
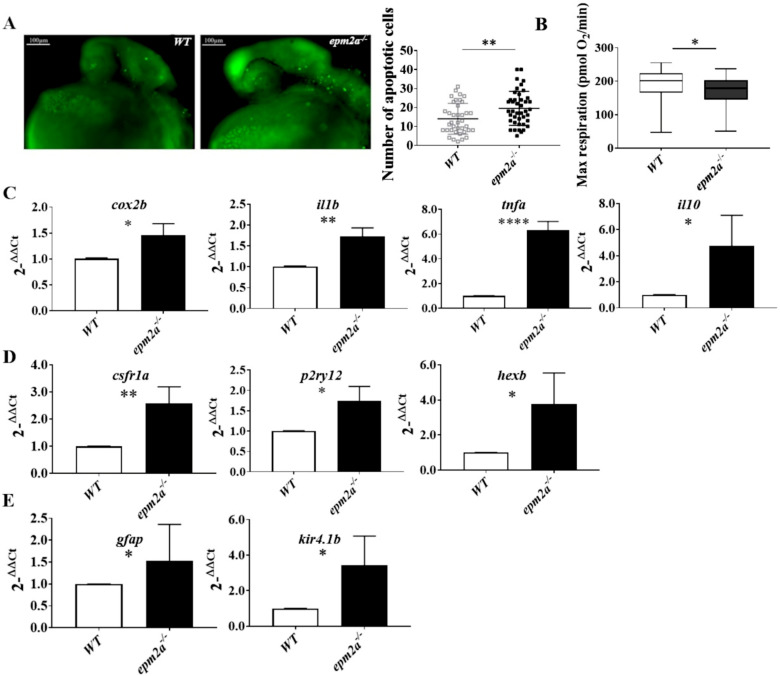
Neuroinflammation, apoptotic death, and mitochondrial dysfunction in *epm2a^−/−^* zebrafish larvae. (**A**) Detection of apoptotic cells by acridine orange staining in controls and *epm2a^−/−^* mutant embryos at 24 hpf (lateral views) alongside a quantitative analysis of apoptotic cells in WT (*n* = 40) and *epm2a^−/−^* (*n* = 44) fish. ** *p* ≤ 0.01, calculated by the Mann-Whitney test. (**B**) Mitochondrial respiratory analysis of controls (*n* = 47) and *epm2a^−/−^* mutant larvae (*n* = 48) at 120 hpf. * *p* ≤ 0.05, calculated by the Mann-Whitney test. (**C**) qRT–PCR analysis of inflammatory and anti-inflammatory cytokines. (**D**) qRT–PCR analysis of microglial genes. (**E**) qRT–PCR analysis of astroglial genes. The mRNA expression levels had been normalized to expression of *β-actin*. Three independent RNA samples (each obtained from about 30–40 larvae) from controls and *epm2a^−/−^* mutant larvae at 120 hpf were analyzed. * *p* ≤ 0.05, ** *p* ≤ 0.01, **** *p* ≤ 0.0001, calculated by Student’s *t*-test. The values are expressed as mean ± SD.

As well as glycogen accumulation, *epm2a^−/−^* larvae also showed changes in other physiological pathways. On studying apoptosis through an in vivo acridine orange (AO) assay, we found an increased number of apoptotic cells in *epm2a^−/−^* embryos compared with WT controls at 24 hpf (Figure 6A). Oxygen consumption rate measurements did not reveal any major impairment of mitochondrial respiratory chain function in *epm2a^−/−^* compared with control larvae at the same stage of development (five dpf), and baseline respiration and ATP production were normal (Supplementary Figure S3). However, *epm2a^−/−^* larvae showed a slight reduction in maximal respiratory capacity compared with controls (Figure 6B).

Furthermore, *epm2a^−/−^* larvae at five dpf showed greater activation of the inflammatory response compared with WT controls as shown by altered transcript levels of all the proinflammatory cytokines studied in qRT-PCR analysis, namely, *cox2b* (*p* < 0.0119), *il1b* (*p* < 0.0018), and *tnfα* (*p* < 0.0001) (Figure 6C). However, upregulation of anti-inflammatory cytokine *il10* (*p* < 0.0258) was also observed (Figure 6C). Analysis of genes specifically expressed in microglia showed upregulation of *p2ry12* (*p* < 0.0101), *hex-b* (*p* < 0.0260) (Figure 6D), and *csf1ra* (*p* < 0.0052) (Figure 6D). Analysis of genes specifically expressed in astrocytes showed the upregulation of *gfap* (*p* < 0.0439) and *kir4.1b* (*p* < 0.0325) (Figure 6E) in laforin-deficient larvae compared with WT controls at five dpf.

### 2.7. Autophagy Impairment and Trehalose Treatment in epm2a^−/−^ Larvae

**Figure 7:**
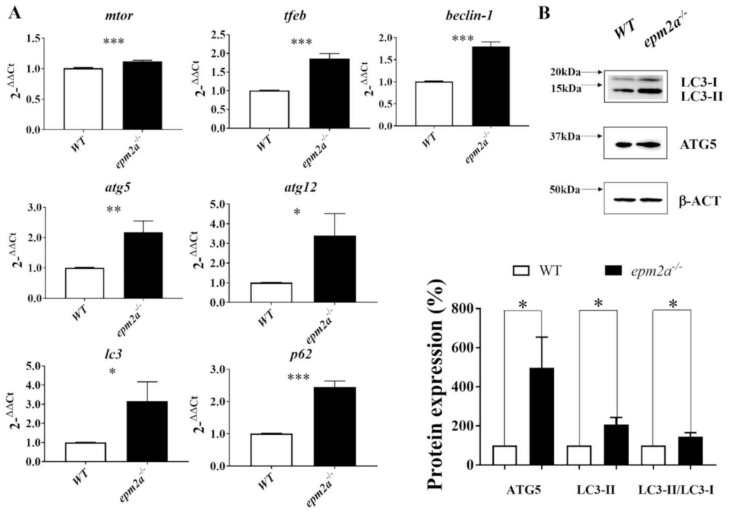
Analysis of autophagy in *epm2a*^−/−^ zebrafish mutants. (**A**) qRT–PCR analysis of autophagy factors (*mtor*, *tfeb*, *beclin-1*, *atg5*, *atg12*, *lc3*, and *p62*) normalized to *β-actin* in *epm2a^−/−^* larvae compared with controls at five dpf. Three independent RNA samples (each obtained from about 30–40 larvae) from controls and *epm2a^−/−^* mutant larvae at 120 hpf were analyzed. * *p* ≤ 0.05, ** *p* ≤ 0.01, *** *p* ≤ 0.001, calculated by Student’s *t*-test. The values are expressed as mean ± standard deviation (SD). (**B**) Three independent larval homogenates from controls (*n* = 50) and *epm2a^−/−^* larvae (*n* = 50) were tested by Western blotting for the expression of ATG5, LC3–I and LC3–II proteins. The levels of the different proteins were normalized to the expression of *β-actin*. * *p* ≤ 0.05 was calculated by Student’s *t*-test.

There is increasing evidence suggesting primary or secondary involvement of autophagy in the neuropathology of LD [16,17,18,21,30,31,45,46]. Therefore, using qRT-PCR, we investigated the autophagy pathway by measuring the transcriptional levels of autophagy regulatory genes *mtor* [47] and *tfeb* [48], of *beclin-1*, which encodes protein important for autophagy initiation [49], and of *atg5*, *atg12*, and *lc3*, which encode proteins important for autophagosome elongation [50]. As shown in Figure 7A, we found the upregulation of genes involved in the autophagy pathway, with slight increases in mtor levels and more pronounced increases in mRNA levels of all the other autophagy factors examined (*tfeb*, *beclin-1*, *atg5*, *atg12*, *lc3*, and *p62*) in *epm2a^−/−^* larvae compared with WT controls at five dpf. We also detected an increased protein level of LC3-II, LC3-II/LC3-I ratio and increased protein level of ATG5 in *epm2a^−/−^* larvae compared with WT controls at five dpf (Figure 7B).

**Figure 8:**
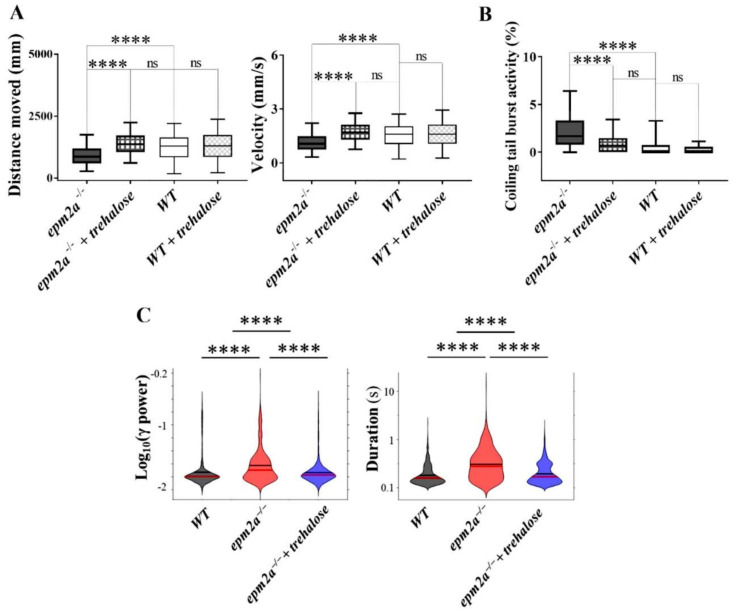
Locomotor, LFP and burst activity recordings after trehalose administration. (**A**) Locomotion analysis at five dpf in controls (WT) treated (*n* = 172) and untreated with trehalose (*n* = 193) and in *epm2a^−/−^* mutant larvae treated (*n* = 213) and untreated with trehalose (*n* = 228). Statistical analysis (**** *p* ≤ 0.0001) was performed using the Kruskal-Wallis test. Dunn’s test was used to perform post-hoc analysis for multiple comparisons after the Kruskal-Wallis test. (**B**) Tail flick analysis performed at 30 hpf in controls (WT) treated (*n* = 91) and untreated with trehalose (*n* = 87) as well as in *epm2a^−/−^* mutant larvae treated (*n* = 106) and untreated with trehalose (*n* = 111). Statistical analysis (**** *p* ≤ 0.0001) was performed using the Kruskal-Wallis test. Dunn’s test was used to perform post-hoc analysis for multiple comparisons after the Kruskal-Wallis test. ns, not significant (**C**) Violin plot of the effect of trehalose on the power and duration of detected events, 11 larvae per group were analyzed at five dpf. Statistical analysis (**** *p* ≤ 0.0001) was performed using the Mann-Whitney test.

Since we found an autophagy impairment in mutant larvae at five dpf, we decided to test whether administration of trehalose, from the early stages of embryonic development (within four hpf) up to five dpf, might at least partially reverse the pathological phenotype of *epm2a^−/−^* larvae. Trehalose is known for its neuroprotective effects [51] and for its capacity to induce autophagy flow [52], although the underlying mechanisms are only partially known [51,53]. Trehalose added to the egg water to reach a final volume of 100 uM was used to treat four hpf larvae. Locomotor behavior (Figure 8A) improved in treated *epm2a^−/−^* larvae compared with untreated mutants, both in terms of distance traveled (*p* ≤ 0.0001) and speed (*p* ≤ 0.0001), suggesting rescue of the motor impairment. The analysis of neuronal hyperexcitability, associated with burst activity, and electroencephalographic activity reached similar results. Indeed, the analysis of 30 hpf tail flick data (Figure 8B) revealed attenuation and reduction of burst activity in trehalose-treated *epm2a* KO embryos (*p* ≤ 0.0001), whereas LFP recordings from five dpf larvae (Figure 8C) showed reduced duration and power of seizure-like events in trehalose-treated *epm2a^−/−^* larvae compared with untreated mutants. Overall, our results indicate that trehalose administered in the early stages of development can reverse the pathophysiological phenotype of *epm2a^−/−^* larvae.

## 3. Discussion

Other than palliative care and the repurposing of a few drugs, current therapeutic approaches in LD include antibody-enzyme fusion (VAL-0417) to degrade LBs [54,55,56], strategies to reduce glycogen synthase [19,57], and new vectors for gene therapy, such as the cationic lipoplexes containing plasmid DNA [58], all developed in vitro or in murine models, and not yet translated into clinical trials. Even in more common monogenic neurodegenerative diseases, there is a growing trend to combine “repair approaches” with therapy based on “downstream approaches” where the aim is not to repair or substitute the defective gene, but rather to focus on experimentally accessible targets in the disease pathogenesis. Thus, identification, with a view to their repurposing, of EMA-/FDA-approved drugs or small molecules or nutrients that act downstream of the gene mutation might accelerate trial readiness in LD. In order to repurpose approved drugs and identify new small molecules for LD, it is necessary to have rapid and efficient screening tools available. In this perspective, we think the zebrafish model of LD could be a valuable and useful aid in drug research.

Loss of laforin function in zebrafish was found to recapitulate the human disease, showing locomotor impairment and neuronal hyperexcitability with spontaneous recurrent seizures (i.e., epilepsy). Indeed, in *epm2a^−/−^* larvae we observed the variability of LFP recordings, identifying spontaneous electrophysiological events and both interictal and seizure-like discharges. This may to an extent reflect what occurs in clinical practice in patients with LD in whom, especially initially, the frequency of the seizures may be relatively low. The course of the disease is progressive and characterized by the increasing frequency and intractability of seizures [59]. The ability of the new zebrafish model to mimic human disease in terms of electrophysiological events makes it an ideal tool for investigating drug response and new opportunities to treat LD.

Furthermore, the *epm2a^−/−^* larvae showed increased glycogen concentration, similar to what is seen in laforin-deficient mice [12], but without evidence of LBs. The absence of LBs has also been seen in *epm2a* KO mice, where polyglucosan aggregation occurs on average at approximately two months of age (or at one month in some LD mouse models) [23], and gradually becomes widespread [60,61,62]. Our data recapitulate major characteristics of the *Epm2a* KO mouse, particularly in its earlier stages of development.

In the zebrafish model of LD, we observed increased apoptotic cell death in mutant larvae compared with controls, in agreement with previous studies showing increased susceptibility to apoptosis of laforin-deficient cells [29], probably caused by the induction of endoplasmic reticulum stress linked to proteasome dysfunction [63]. In addition, laforin appears to have a possible anti-apoptotic function [64]. It is possible that increased susceptibility to apoptosis contributes to neurodegeneration in LD. This apoptotic death occurs in zebrafish larvae independently of the formation of LBs. “Dark cell death”, a unique form of non-apoptotic cell death that precedes the formation of LBs, has been described in other models of LD [60,65]; indeed, many degenerated neurons do not show visible LBs [60,65]. These data suggest multiple causes of neuronal death in LD.

We did not observe any major impairment of mitochondrial bioenergetics, although *epm2a^−/−^* larvae at 5 dpf seemed to show a weak response to stress associated with increased energy demand when more ATP is required to maintain cellular function. In contrast, increased oxidative stress is a feature of different models of LD [22], and could be related to the alteration of intracellular proteolytic systems—and thus of mitophagy [32]—but also to mitochondrial dysfunction [22] and to the reduction of enzymes involved in ROS detoxification [22]. We cannot exclude that oxidative stress could become evident at later developmental stages in *epm2a^−/−^* zebrafish larvae and contribute to cell death.

As seen in KO mice [23,24], we observed the activation of inflammatory responses in *epm2a^−/−^* larvae. Analysis of the inflammatory genes in our model suggests that microglial populations with different phenotypes may coexist in the early stages of the disease. The upregulation of pro-inflammatory cytokines genes (*tnfα*, *cox2b* and *il1b*) and of *csfr1a* suggests a pro-inflammatory activation of microglia, leading to a detrimental phenotype, sometimes called disease-associated microglia (DAM), which is described in several neurodegenerative diseases [66,67]. CSF1R has been found to be increased in disease-associated microglia in the Alzheimer’s disease brain [68]. Upregulation of the CSF1/CSF1R pathway may, via a positive feedback mechanism, help to support the DAM signature [66,69]. The early activation of neuroinflammation observed in the pathophysiology of our laforin-deficient zebrafish model recalls other neurodegenerative disorders characterized by intracerebral accumulations [70] and suggests new targets for alleviating symptoms in preclinical studies. On the other hand, the upregulation of anti-inflammatory cytokines genes (e.g., *il10*) suggests that some microglial cells, at least, have an anti-inflammatory phenotype [71]. We also observed the upregulation of the purinergic receptor gene (e.g., *p2ry12*), whose density is modified depending on the state of microglial activation [72]; the first step of microglial activation in response to chemo-attractive signals released by damaged cells [73,74] is stimulation of P2RY12, subsequently followed by its downregulation [75]. We analyzed the homeostatic gene *hexb*, a lysosomal hydrolase regulating the metabolism of gangliosides and involved in autophagy [76], whose expression does not generally change in the transition between homeostatic and harmful microglia [77,78]. The *hexb* upregulation we observed in laforin-deficient larvae supports the possibility that dysregulation of lysosomal function and autophagy in microglia may contribute to the neuropathology of LD [16,17,18,21,30,31,45,46]. These results are also in line with those described in microglia from a mouse model of Alzheimer’s disease [79]. Expression analysis of some astrocytic markers showed upregulation of *gfap* and *kir4.1b* genes in laforin-deficient larvae at five dpf, suggesting a role for these cells in the *epm2a^−/−^* zebrafish model; early astrocytic activation has also been observed in mouse models of the disease [23,24,80]. Moreover, we previously observed the upregulation of *kir4.1b*, which is associated with autism-epilepsy phenotypes in humans [81], and we cannot exclude its possible participation in the epileptic phenotype observed in *epm2a^−/−^*. Indeed, both upregulation and downregulation of Kir4.1 seem likely to produce neuronal hyperexcitability [82,83]. It must be underlined that zebrafish possess no star-shaped glial cells, but rather radial glia [84,85] whose functions have yet to be precisely defined. It seems, however, that these glial cells carry out many of the functions of classical mammalian astroglia, such as neurogenesis and homeostatic roles in neural circuits and brain barriers [85]. The involvement of this cell type has also been described in other neurodegenerative diseases modelled in zebrafish, in which astrogliosis [86] or induction of neuronal regeneration [87] has been observed. However, the possible role of glial cells in LD and their role in teleost fish compared with mammals remain to be studied in more detail.

Given the evidence pointing to primary or secondary involvement of the autophagy pathway in LD [16,17,18,21,30,31,45,46], our study focused on the upregulation of several autophagy genes and on increased protein levels of ATG5, LC3-II and the LC3-II/LC3-I ratio from early development. These results could indicate a compensatory increase in autophagic flux [88], similar to what has been observed in Batten disease [89], another form of PME. This compensation could explain in part the absence of LBs at this stage of the disease, with ensuing loss of autophagic compensation as the disease progresses and LBs become evident. As an alternative hypothesis, there might occur a decreased flux and the subsequent accumulation of LC3 due to a block in fusion or degradation, as documented by increased LC3-II [88] and increased LC3-II/LC3-I ratio [90], followed by a compensatory increase upstream. More robust studies in the new model of LD are warranted.

Early dietary supplementation with the autophagy flux modulator trehalose ameliorated PTZ-induced seizure susceptibility in laforin-deficient mice [38] without acting on LBs. In zebrafish we corroborated these data: Early administration of trehalose reduced neuronal excitability and the frequency of spontaneous seizures and rescued the motor impairment, offering further support to its clinical use in children with LD. The mechanism by which trehalose induces autophagy is still unknown, but it appears to act as an inducer of autophagy flux and lysosomal biogenesis by activating TFEB [52]. In our laforin-deficient larvae we observed high levels of *tfeb* mRNA, which has also been documented in other neurodegenerative diseases [91]. The increased *tfeb* expression could be a sign of a possible compensatory mechanism activated to overcome the impaired lysosomal function [92] through autophagy induction. It is also possible that TFEB is sequestered in the cytosol in the inactive form and is therefore unable to act at the nuclear level to stimulate autophagy genes. In fact, our model also showed the upregulation of *mtor*, which phosphorylates TFEB and blocks it in the cytosol in the inactive form [93]. Our hypothesis is that trehalose may act by activating TFEB and promoting its translocation to the nucleus [52]. Trehalose also appears to act on autophagy in an mTOR-independent manner [53], favoring its chaperone function [53], preventing inflammatory responses [94], and inducing de novo autophagosome formation [95]. Given the good safety profile of trehalose in children and adults, and the multiple ongoing clinical trials of the treatment (ClinicalTrials.gov; Available online: https://clinicaltrials.gov/ct2/results?cond=trehalose&term=&cntry=&state=&city=&dist=; accessed 3 June 2022), it is tempting to anticipate its use in LD patients before too long.

In conclusion, the laforin-deficient zebrafish is a valuable new model for the study of LD, recapitulating the main features described in the mouse models and patients (Supplementary Table S1), providing a unique platform for studying the early stages of the disease and for more robust and cheap drug screening. Further research will be needed on adult fish, since the nervous system of larvae is incomplete and can limit the translation of complex neurological disorders. However, we believe that *epm2a^−/−^* larvae will likely help to speed up the drug discovery studies in vivo ahead of more expensive studies in mice.

## 4. Materials and Methods

### 4.1. Zebrafish Maintenance

Experiments were conducted on *epm2a^−^/^−^*; *mitfa^−/+^*; *Tg(neurod1:GCaMP6F)* F2 lines which will henceforth be referred to as *epm2a^−^/^−^* and an *mitfa^−/+^*; *Tg (neurod1:GCaMP6F)* strain which will henceforth be referred to as WT. Before proceeding with *mitfa^−/+^*; *Tg(neurod1:GCaMP6F)*, we carried out behavioural analyses to test any differences from the WT-AB strain. Analysis of the locomotor behaviour of *mitfa^−/+^*; *Tg(neurod1:GCaMP6F)* and WT-AB showed no differences between the two strains and is shown in Supplementary Figure S4. *epm2a^−^/^−^* experimental fish are generated from intercrossing *epm2a^−/−^* males and females.

Adults were housed in tanks at a density of no more than five zebrafish per liter at a constant temperature of 28 °C with a 14-h light/10-h dark cycle. Zebrafish eggs and embryos were collected and grown at 28.5 °C in egg water (60 µg/mL of “Instant Ocean”, Sea Salts, (Aquarium Systems, Sarrebourg, France)) and E3 medium [292.2 mg NaCl, 12.6 mg KCl, 48.6 g mM CaCl2 2H2O, and 39.8 mg MgSO4, per 1 L of deionized water] respectively using established procedures [96] and staged in either hours post fertilization (hpf) or days post fertilization (dpf). All compounds used for E3 medium solution preparation were purchased from Sigma (St. Louis, MO, USA). The generation of the mutant, using CRISPR/cas9 technology, was performed with the ethical approval of the Italian Ministry of Health (approval n° 258/2021-PR) in accordance with the European Union Directive 2010/63/EU on the protection of animals used for scientific purposes, under the supervision of the Institutional Animal Care and Use Committee of the University of Pisa, and in compliance with the 3R principles.

### 4.2. Establishing Mutant Lines

The selected sgRNA was chosen among the top targets identified by CHOPCHOP software (CHOPCHOP. Available online: www.chochop.rc.fas.harvard.edu/index.php; accessed 8 January 2022) set with NGG PAM sites and zero predicted off-targets (fewer than three mismatches in the *epm2a*-targeting 20-mer). The sgRNA was designed against exon two of the *epm2a* transcript (ENSDARG00000059044) (Supplementary Table S2) and generated as already described [97]. The sgRNA was transcribed using the Megascript T7 Transcription Kit (Invitrogen, Heidelberg, Germany). The optimized Cas9 mRNA, for genome editing in zebrafish, was transcribed from a linearized template plasmid pCS2-nCas9n using the mMESSAGE mMACHINE™ SP6 Transcription Kit (ThermoFisher Scientific, Waltham, MA, USA). RNA concentration was quantified using a NanoDrop spectrophotometer (Optosky, Xiamen, China) and diluted to 500 ng/µL. About 100 ng of *epm2a*-sgRNA and 500 ng of Cas9 mRNA were co-injected into 1-cell stage embryos to ensure high-efficiency delivery of the injected mRNA to the embryo. The volume of solution injected was ~1 nL. At least three independent injection experiments were performed with spawns from different founder fish to control for the batch effect. CRISPR/Cas 9 was into wild-type (WT) AB genotype.

### 4.3. Genotyping

For mutation screening, sgRNA-injected F0 embryos, raised to adulthood and outcrossed with the *Tg(neurod1:GCaMP6F)* strain in the nacre *(mitfa^−/+^)* background (kindly donated by Claire Wyart from Institut du Cerveau et de la Moelle Épinière, Paris, France) [98], were used for the study to obtain F1 heterozygous embryos. Adults potentially carrying mutations were identified by PCR and fragment analysis using genomic DNA from 16 randomly selected F1 embryos. The sequence of the primers used is listed in Supplementary Table S2. F1 heterozygous fish carrying a 5-bp insertion mutation in the targeted site were selected and inter-crossed to generate the F2 homozygous *epm2a^−/−^* line.

### 4.4. In Situ Hybridization

Whole-mount in situ hybridization was performed in 24 hpf WT embryos as described previously [99]. The sequences of the primers used can be found in Supplementary Table S3.

### 4.5. Analysis of Larval Morphology

Live zebrafish were mounted on glass depression slides with 1% low-melting agarose. Images were obtained using a Leica M205FA stereomicroscope (Leica Microsystem, Wetzlar, Germany). Zebrafish body length was measured using Danioscope software (Noldus Information Technologies, Wageningen, The Netherlands) [100].

### 4.6. Quantitative (q)RT-PCR

Gene expression levels of *epm2a* mRNA were examined using RNA pooled from WT and *epm2a^−/−^* larvae. Total RNA was extracted from 30 larvae at 120 hpf using the Quick RNA Miniprep Kit (Zymo Research, Irvine, CA, USA) according to the manufacturer’s instructions and quantified with a NanoDrop™ ND-1000 spectrophotometer (Thermo Scientific, Waltham, MA, USA). Extraction of cDNA and qRT-PCR were performed as described elsewhere [101]. Relative expression levels of each gene were calculated using the 2^−ΔΔCT^ method [102]. The results obtained in at least three independent experiments were normalized to the expression of the housekeeping gene, *β-actin* (ENSDARG00000037746). The expression analysis of *epm2a* mRNA in mutant larvae was calculated setting the mean of the controls at one, and the *p*-value was calculated using GraphPad Prism 6 software (GraphPad Software, Inc., San Diego, CA, USA). The primers used are listed in Supplementary Table S4.

### 4.7. Western Blotting

Larvae collected at 120 hpf were lysed in RIPA buffer supplemented with 1 mM PMSF, 1 mM sodium fluoride, and 1 mM sodium vanadate. Embryo proteins (about 50 μg) were electrophoresed in 10% SDS-PAGE gel and transferred to nitrocellulose membranes. Western blotting was performed as previously described [103,104]. The primary antibodies used were mouse anti-β-ACTIN (GTX629639, GeneTex, Irvine, CA, USA, 1:2500), rabbit anti-LC3 (NB100-2220, Novus Biologicals, Centennial, CO, USA, 1:1000), and rabbit anti-ATG5 (NB110-53818, Novus Biologicals, 1:500), whereas the secondary antibodies were peroxidase-conjugated anti-mouse and anti-rabbit (Cell Signaling Technology Inc., Danvers, MA, USA).

### 4.8. Glycogen Assay

The amount of glycogen was determined in dechorionated zebrafish larvae (minimum 10 per experiment) at five dpf, using the Glycogen Assay Kit II (Abcam, Cambridge, UK) according to the manufacturer’s instructions and as previously described [105].

### 4.9. Histological Preparation for Periodic Acid-Schiff (PAS) Staining

Zebrafish larvae at 120 hpf were sacrificed by overdose of anesthesia (0.25 mg/mL, MS-222, Sigma-Aldrich, St. Louis, MO, USA) and fixed in cold 10% neutral buffered formalin at 4 °C overnight. Prior to processing, to optimize sectioning, the larvae were embedded in 1% agarose blocks, as previously described [106]. Tissue processing of agarose blocks was then performed in a controlled automatic processor (Shandon TP 1020, Leica Microsystem), followed by paraffin embedding. Paraffin blocks were sliced into 5 μm sections and stained with periodic acid-Schiff (PAS) stain under standard protocol. Sections were then examined using a light microscope (Eclipse 80i, Nikon, Tokyo, Japan).

### 4.10. Immunohistochemistry Staining of Whole-Mount Zebrafish Embryos

To prevent the development of pigmentation, embryos were treated with 0.005% phenylthiourea from 24 hpf. Whole-mount immunohistochemistry was performed in 48 or 120 hpf embryos fixed in 4% paraformaldehyde (PFA) overnight at 4 °C and stored in methanol as described in Kani, et al. [107]. The primary antibody used was mouse anti-Syt2 (ZNP-1) (ab154035, Abcam, 1:250). The secondary antibodies used was peroxidase-conjugated anti-mouse (Cell Signaling Technology Inc., Danvers, MA, USA). Images were acquired using the Leica M205FA microscope (Leica, Wetzlar, Germany).

### 4.11. Detection of Apoptotic Cells

To visualize apoptotic cells, in vivo staining was carried out in live, PTU-treated, manually-dechorionated larvae at 24 hpf using the vital dye acridine orange (AO, #235474; Sigma-Aldrich). Zebrafish embryos were incubated with 10 μg/mL AO solution for 15 min in the dark; the larvae were then washed three times with E3 medium. We counted AO-positive cells within a pre-defined area and a quantitative analysis was performed as described elsewhere [100,108].

### 4.12. Mitochondrial Respiratory Analysis

Mitochondrial respiration was analyzed in control (WT) and homozygous *epm2a^−/−^* larvae at 120 hpf using the XF24 extracellular flux analyzer (Seahorse Bioscience, North Billerica, MA, USA). The dual analyte sensor cartridges were soaked in an XF calibrator solution (Seahorse Bioscience) and placed in 24-well cell culture microplates overnight at 28 °C to hydrate. About 30 min before the trial period, the injection ports on the sensor cartridge were filled with oligomycin (0.6 μmol/L), FCCP (6 μmol/L), and rotenone (5 µM) plus antimycin A (0.182 μmol/L). The 120 hpf larvae were staged and placed in 20 of the 24 wells of an islet microplate. The islet plate acquisition screens were placed on the measurement area to hold the larvae in place. Four wells were left empty as a control. Each well was filled with 500 µL of E3 medium (pH 7.4). A standard approach was used to measure basal respiration, ATP production, maximal respiration rate, and spare respiratory capacity [109].

### 4.13. Locomotor Behavior

Locomotor behavior (distance traveled and velocity) was measured in homozygous *epm2a^−/−^* F2 larvae and WT at five dpf using the Daniovision device (Noldus Information Technology, Wageningen, The Netherlands). Briefly, single larvae were transferred into 96-well plates containing 300 μL of E3 medium per well. Then, one plate at a time was placed in the DanioVision system, and larval locomotor activity was recorded for 30 min and analyzed using EthoVision XT software (Noldus Information Technology, Wageningen, The Netherlands). Coiling behavior was measured to assess burst activity in homozygous *epm2a^−/−^* and WT embryos at 30 hpf; to this end, the number of tail flicks was measured in 30-s time frames using Danioscope software (Noldus Information Technology, Wageningen, The Netherlands).

### 4.14. LFP Recordings and Analysis

Electrophysiological forebrain recordings were performed in 120 hpf larval zebrafish to characterize the epilepsy phenotype. The larvae were placed on a drop of 1.2% low-melting point agarose and LFPs were recorded with an AgCl electrode inside a glass micropipette backloaded with 2M NaCl. Electrophysiological signals were amplified 500-fold (EXT-02F, NPI, Tamm, Germany), band pass filtered (0.3–1300 Hz), and digitized at a rate of 5 kHz (Axon Digidata 1550B, Clampex 10.7.0.3, Molecular Devices, Berkeley, CA, USA). The microelectrode was positioned by advancing its tip until it punctured the skin, and then it was carefully advanced into the forebrain [110]. Data analyses of the LFP recordings were performed using a suite of custom Matlab scripts. Burst activity analysis was performed on the LFP signals filtered in the 30–95 Hz band as already described [111]. The root mean square (RMS) power of the signal was computed on the recording on rolling windows of 250 ms and in steps of 50 ms. The distribution of the logarithm of the RMS power measurements was fitted with a single gaussian distribution. In some recordings, the distribution was characterized by an asymmetry due to a high-energy population. Those distributions were fitted with double gaussians56, then the ROC curves and their area under the curve (AUC) were calculated; values > 0.9 were considered acceptable and events were extracted as described in Supplementary Figure S5. For normally distributed recordings, bursts were identified by extracting all events with a power higher than three standard deviations (SD) from the mean. Violin plots were produced using the online-available function ‘violin.m’ [112]. Statistical analysis was performed using GraphPad Prism version 9 software. (GraphPad Software, San Diego, CA, USA).

### 4.15. Pharmacological Treatments

Fifty mutant and fifty WT 4 hpf embryos were randomly placed in 60 mm × 15 mm Petri dishes containing trehalose diluted in egg water. Trehalose stock solutions were prepared in Milli-Q water (Merck-Millipore, Milan, Italy) and diluted in egg water to the final administered concentrations. The 100 µM concentration was chosen as the working dilution. To test treatment efficacy, we performed tail coiling and locomotor behavioral assays and LFP recordings.

### 4.16. Statistics

All data in the manuscript represent three or more independent experiments. Statistical analysis was performed using GraphPad Prism 6. All quantitative variables were analyzed applying either parametric or non-parametric methods, depending on the distribution shown by the Shapiro-Wilk test. For multiple comparisons (post-hoc analysis), Dunn’s test was performed after the Kruskall-Wallis test, since the data of the various groups examined did not follow the gaussian distribution. Statistical significance is reported as: * *p* ≤ 0.05, ** *p* ≤ 0.01, *** *p* ≤ 0.001, or **** *p* ≤ 0.0001.

## Data Availability

Raw data and images are available upon request from the corresponding author.

## Supplementary Materials

The following supporting information can be downloaded at: https://www.mdpi.com/article/10.3390/ijms23126874/s1.
