# Supplementary material for: Trehalose Treatment in Zebrafish Model of Lafora Disease
Source: Int J Mol Sci. 2022 Jun 20;23(12):6874. doi: 10.3390/ijms23126874 (PMC9224929; doi:10.3390/ijms23126874)
Supplement: Supplementary file 1 [file ijms-23-06874-s001.zip › ijms-1762414-supplementary.pdf]

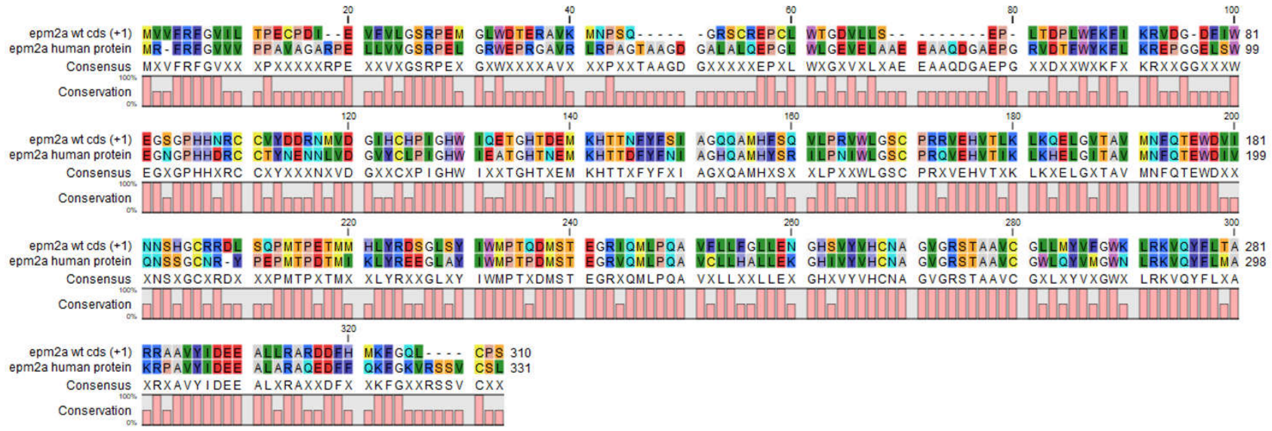

Figure S1. Alignment of human and zebrafish epm2a protein.

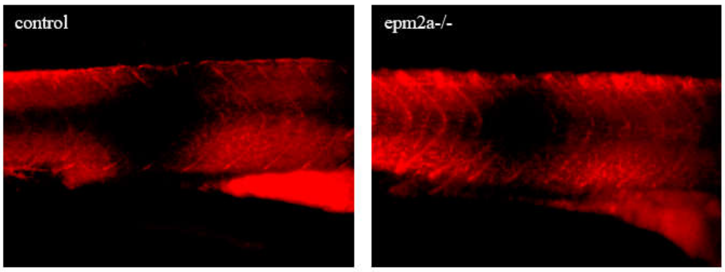

Figure S2. Immunocytochemistry with motor neuron marker Syt2 showed no morphological alterations in *epm2a*$^{-/-}$ larvae compared with wild-type control at five dpf.

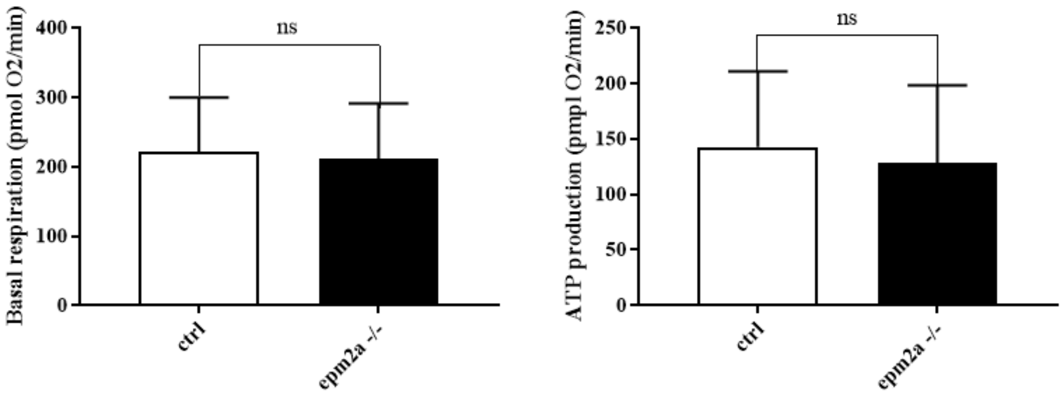

Figure S3. Mitochondrial respiratory analysis of controls (n = 47) and epm2a-/- mutant larvae (n = 48) showed no difference in baseline respiration and ATP production. Statistical analysis was calculated using a Student's t-test.

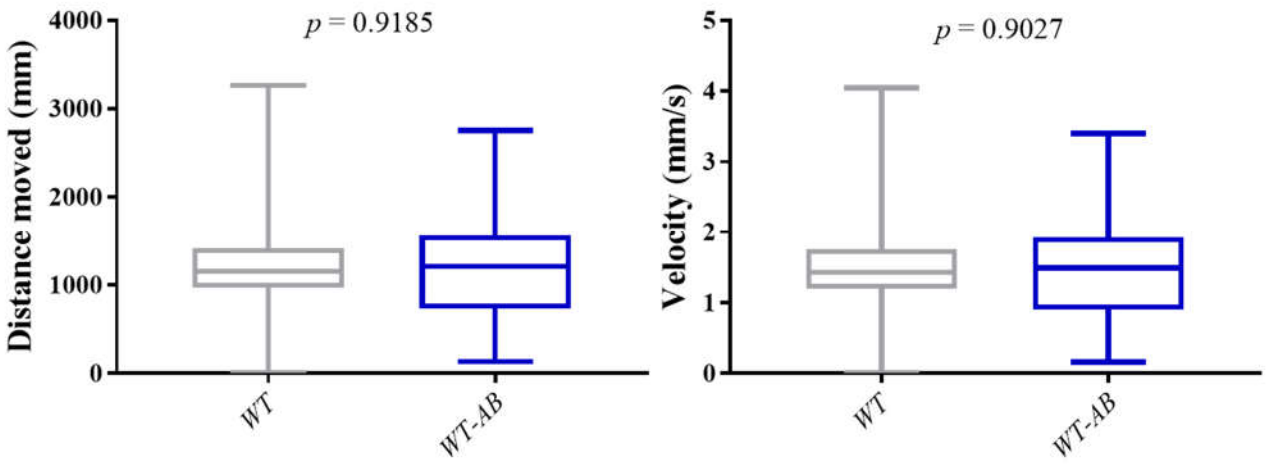

Figure S4. Locomotor analysis. Automated analysis of spontaneous motor activity revealed no differences in distance travelled and velocity between *mitfa*$^{-/+}$; *Tg (neurod1:GCaMP6F)* (WT) (n=152) compared with WT-AB (n=85) at 120 hpf. Statistical analysis was performed using the Mann-Whitney test.

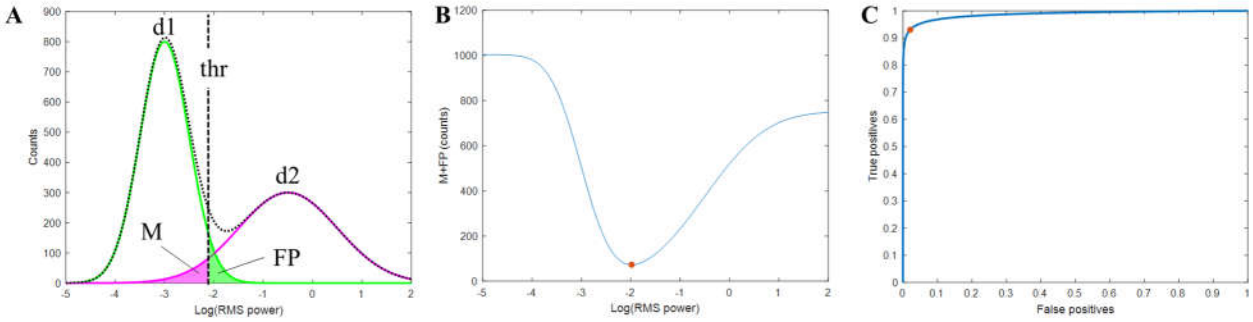

Figure S5. Analysis pipeline for the detection of high-energy events. (**A**) Distribution of the Log of the gamma power (black dotted line). The low-energy mode and high-energy mode (d1 and d2, respectively) are plotted in green and magenta. At a certain threshold (thr), we identify M (misses) and FP (false positives) as $\int_{-\infty}^{thr} d2(x)\,dx$ $\int_{-\infty}^{thr} d2(x)\,dx$ and $\int_{thr}^{+\infty} d1(x)\,dx$ $\int_{thr}^{+\infty} d1(x)\,dx$ respectively (integrals on discrete arrays are computed with the Matlab function 'trapz'). (B) To choose the threshold for identifying high-energy events, we define and minimize the error function $err = M + FP, err = M + FP$, where err, M, and FP are a function of thr. The threshold is set as the minimum of err, indicated with a red dot. (C) ROC function. In this example, the area under the curve (AUC) is 0.9872. The position on the ROC curve for the chosen thr is indicated with a red dot.

Table S1. Comparing traits of zebrafish, mouse and human patients with *EPM2A* mutations.

	**Human**	**Mouse**	**Zebrafish**
**Gene**	*EPM2A*	*Epm2a*	*epm2a*
**Mutation**	Over 50 mutations described, the most common mutation is the p.R241X	*Epm2a*-KO mice, several double-KO mice, and recently, the first laforin-R240X knock-in mouse model was developed	*epm2a*-KO ZF (p.Thr54Asnfs*74)
**Neuropathological features**	Glycogen accumulation, Lafora bodies, neuronal death, impaired autophagy, oxidative stress	Glycogen accumulation, Lafora bodies, neuronal cell death, impaired autophagy, oxidative stress and increased neuroinflammation	Glycogen accumulation, increased brain apoptosis, impaired autophagy, increased neuroinflammation
**Phenotype**	Progressive drug-resistant myoclonus and seizures, cerebellar ataxia and dementia	Impaired behavioral responses, ataxia, spontaneous myoclonic seizures or PTZ-induced seizures and EEG epileptiform activity	Locomotor impairment and spontaneous seizures
**References**	1-2-4-5-15-21-22-28-29-32-33	12-13-16-21-22-23-24-29-34-45-46-60-61-62	This study

Table S2. sgRNA sequence and primers for genotyping.

epm2a-sgrna1	5′-TAATACGACTCACTATAGGAGCCGTGCCTGTGGACCGGTTTTAGAGCTAGAAATAGC-3′
epm2a-1F	TCATGGCCAAAACCCCTCTAAAAC
epm2a-1R	GCCCACCACTAATATATACCAAAAGT

Table S3. Primers used for the whole-mount in situ hybridization.

epm2a-ish-f	GTTTAAGTTCATAAAGCGGGTCGAT
epm2a-T3-ish-f	AATTAACCCTCACTAAAGGGGTTTAAGTTCATAAAGCGGGTCGAT
epm2a-ish-r	CAGAGCTGTCCAAACTTCATATGAA
epm2a-T3-ish-r	AATTAACCCTCACTAAAGGGCAGAGCTGTCCAAACTTCATATGAA

Table S4: qPCR primers used for gene expression analysis.

Gene	Nucleotide sequence
epm2a-Fw	TGTTCAGGTTTGGTGTTATTTTGAC
epm2a-Rv	ATCCACCATGTTTCTGTCATCATAC
tfeb-Fw	AAGAAAGACAACCACAACCTGATT
tfeb-Rv	ACATCCTTCTGCATGCGTTTAATA
beclin-1 Fw	GGCTTTCCTTGACTGTGTCC
beclin-1 Rv	CCTTTGTCCACATCCATTCTG
mtor-Fw	TTATCGTGCTGGTCCGAGCT
mtor-Rv	AAGTGGGCCCTTATCGCTGT
atg5-Fw	AGAGAGGCAGAACCCTACTATC
atg5-Rv	CCTCGTGTTCAAACCACATTTC
atg12-Fw	TTCATCTCACGCTTCCTCAA
atg12-Rv	CGTCACTTCCGAAACACTCA
lc3a-Fw	CGAGTCGACCGACAATTTAGC
lc3a-Rv	TCCTTGCAACGATCAGCGAA

p62-Fw	CGATGTTTTTGTCGGTCTCA
p62-Rv	CAAGAGCCAAACCCATCATT
cox2b-Fw	CCCTGTCAGAATCGAGGTGT
cox2b-Rv	TTGGGAGAAGGCTTCAGAGA
tnfa-Fw	GGGCAATCAACAAGATGGAAG
tnfa-Rv	GCAGCTGATGTGCAAAGACAC
il1b-Fw	GGACTTCGCAGCACAAAATGAA
il1b-Rv	TTCACTTCACGCTCTTGGATGA
il10-Fw	CTTTAAAGCACTCCACAACCCCAA
il10-Rv	CTTGCATTTCACCATATCCCGCTT
gfap-Fw	GCTCTGAGACAAGCGAAGCA
gfap-Rv	CACGGAGAGATTCCAGGTTCA
kcnj10b-Fw	GCGGCGAGACGATTCGCTTCA
kcnj10b-Rv	CTTCCCCGTCACCTGGCAACC
hexb-Fw	CTTTGGGGAGAGTATGTGGACGC
hexb-Rv	CAGGTATGCCTCTCCTGACCAT
p2ry12-Fw	CTTCAGGTCGTCGCTGTTTA
p2ry12-Rv	AGTGCGTTTCCCTGTTGAT
csf1ra-Fw	CCTGATCCGCAACGTTCATCCT
csf1ra-Rv	GCTTTGGGCAGCATTCTTGAGG
bactin2-Fw	GCAGAAGGAGATCACATCCCTGGC
bactin2-Rv	CATTGCCGTCACCTTCACCGTTC
